# Nickel Bioaccumulation in Oral Biofilm, Gingival Tissue, and Saliva During Fixed Orthodontic Treatment: A 12-Month Prospective Cohort Study on Predictors and Salivary pH Correlation

**DOI:** 10.4317/jced.62923

**Published:** 2025-08-01

**Authors:** Lizeth Galviz-García, Sandro Romero-Romero, Alejandra Herrera-Herrera, Jairo Mercado-Camargo, Antonio Díaz-Caballero, Jaime Plazas-Román, Carlos M Ardila

**Affiliations:** 1 Orthodontics, Universidad de Cartagena, Bolívar, Colombia; 2 Master in Pharmacology. Professor, Metropolitan University, Barranquilla, Colombia; 3 PhD in Biomedical Sciences, Universidad de Cartagena. Professor, Universidad de Cartagena, Cartagena, Bolívar, Colombia; 4 PhD in Biomedical Sciences, Universidad de Cartagena. Leader of the research group GITOUC. Professor, Universidad de Cartagena, Cartagena, Bolívar, Colombia; 5 Master in Bioinformatics. Professor, Universidad de Cartagena and Universidad del Sinú, Cartagena, Bolívar, Colombia; 6 PhD. Department of Periodontics, Saveetha Dental College and Hospital, Saveetha Institute of Medical and Technical Sciences, Saveetha University, Chennai, Tamil Nadu, India; 7 Basic Sciences Department. Biomedical Stomatology Research Group, Faculty of Dentistry Universidad de Antioquia, UdeA, Medellín, Colombia

## Abstract

**Background:**

Nickel-based alloys in fixed orthodontic appliances are susceptible to electrochemical corrosion in the oral environment, releasing ions with cytotoxic and allergenic potential. While previous studies have documented initial nickel release patterns, the longitudinal dynamics of its accumulation across oral biospaces (saliva, biofilm, gingival tissue) and interactions with salivary pH remain poorly characterized. This study investigates nickel accumulation in saliva, oral biofilm, and gingival tissue over 12 months of orthodontic treatment and its association with salivary pH.

**Material and Methods:**

This 12-month prospective cohort study enrolled 120 participants undergoing fixed orthodontic treatment. Nickel concentrations were quantified via graphite furnace atomic absorption spectrophotometry in three biospaces at baseline (T0), 6 months (T1), and 9–12 months (T2). Salivary pH was measured concurrently. Statistical analyses included non-parametric longitudinal comparisons, inter-biospace contrasts, Spearman correlations, and multivariate regression modeling to identify predictors of nickel accumulation.

**Results:**

Nickel exhibited distinct spatiotemporal patterns: progressive accumulation in biofilm (0.0008→21.5833 µg/L, *p*<0.001) versus biphasic kinetics in gingiva and saliva (peaking at T1 then declining). By T2, biofilm concentrations were 1000× higher than other biospaces (*p*<0.001). Treatment duration was the strongest predictor across all compartments (β=0.56–0.67, *p*<0.001), with biofilm accumulation additionally modulated by salivary pH (β=0.22, *p*=0.027) and age (β=−0.19, *p*=0.041). A time-dependent correlation emerged between salivary pH and biofilm nickel (T0: r=0.17, *p*=0.112; T2: r=0.41, *p*=0.008), suggesting pH-microbiome interactions.

**Conclusions:**

Oral biofilm serves as the dominant long-term nickel reservoir during orthodontic treatment, with accumulation dynamics influenced by treatment duration, pH, and age. The pH-dependent biofilm-metal interaction highlights its potential as a biomarker for exposure monitoring and a target for preventive strategies. These findings support the development of pH-modulating oral care protocols and corrosion-resistant orthodontic materials to mitigate nickel exposure risks.

** Key words:**Nickel, saliva, Spectrophotometry, Atomic, Orthodontic Appliances, Hydrogen-Ion Concentration.

## Introduction

Fixed orthodontic appliances are indispensable in modern dentistry, relying heavily on metal alloys for their mechanical properties, such as shape memory and superelasticity [1,2]. Among these alloys, nickel (Ni) is a fundamental component, yet its prolonged exposure to the dynamic oral environment raises concerns regarding biological stability and the potential consequences of ion release [3,4].

The oral cavity presents a uniquely challenging environment for biomaterials, characterized by fluctuating pH, temperature variations, electrolyte concentrations, and microbial activity [5,6]. These conditions promote electrochemical corrosion and biodegradation of metal alloys, with pH playing a particularly critical role in accelerating surface degradation [7,8]. Consequently, nickel ions released from orthodontic appliances may redistribute across multiple oral compartments—including saliva, biofilm, and gingival tissues—raising questions about their accumulation patterns and biological effects.

Nickel-titanium (NiTi) alloys typically contain ~55% nickel, while stainless steel brackets and bands incorporate 6–12% [9,10]. Although these alloys develop passive oxide layers that theoretically limit ion release, clinical studies confirm that nickel ions are progressively liberated under oral conditions [11]. This is concerning given nickel’s status as a potent allergen, capable of inducing type IV hypersensitivity reactions [12]. Clinically, nickel accumulation has been linked to adverse biological responses, including gingival overgrowth during orthodontic treatment [13]. *In vitro* and *in vivo* studies further associate chronic nickel exposure with reduced cell viability, DNA damage, and oxidative stress [14,15].

Recent investigations suggest a biphasic release pattern: an initial surge post-placement, followed by a gradual decline due to surface passivation [16]. However, critical gaps remain in understanding the differential distribution of nickel across oral biospaces and its long-term accumulation kinetics. Of particular interest is oral biofilm, which may act as a biological reservoir for metal ions [17,18]. Microbial communities can exacerbate corrosion through metabolic activity while concentrating nickel ions via interactions with exopolysaccharides and surface proteins [19].

This study seeks to characterize the temporal patterns of nickel accumulation in saliva, biofilm, and gingival tissue over the course of orthodontic treatment, while evaluating the influence of salivary pH and treatment duration on ion retention.

## Material and Methods

- Study Design and Participant Selection

This prospective cohort study followed 120 participants (aged 14–40 years) over a 12-month period during fixed orthodontic treatment. A total of 1,080 samples—comprising saliva, supragingival biofilm, and gingival epithelial cells—were collected at three time points: baseline (T0, before appliance installation), 6 months (T1), and 9–12 months post-installation (T2). The sample size was determined via power calculation based on prior data (9), ensuring 95% confidence, 80% statistical power, and the ability to detect clinically meaningful differences of 1.5 μg/L (assuming a standard deviation of 0.9 μg/L).

Participants were included if they underwent treatment with conventional metallic fixed appliances from a single manufacturer. Exclusion criteria comprised pre-existing oral metallic structures (e.g., prostheses or implants), residence near industrial zones, smoking, systemic diseases, or any condition potentially confounding metal exposure.

This study’s protocol received approval from the Bioethics Committee of the Faculty of Dentistry at the Universidad de Cartagena, Colombia. All participants provided informed consent by signing a consent form before enrolling in the study. Moreover, the guidelines of the Declaration of Helsinki were followed throughout the research.

- Sample Collection and Handling

Saliva: Unstimulated saliva (5 mL) was collected after participants refrained from eating or oral hygiene for 2 hours. Following a distilled water rinse and a 5-minute rest, samples were immediately refrigerated at 4°C to preserve stability.

Supragingival Biofilm: Using a sterile microapplicator, supragingival biofilm was systematically scraped from the buccal cervical region of the upper right first molar. Five horizontal strokes covered a standardized 3 mm² area, with the collected material transferred to phosphate-buffered saline (PBS; 0.5 mL).

Gingival Epithelial Cells: A Cytobrush was rotated 10 times on the buccal mucosa adjacent to the upper right first molar to harvest epithelial cells. The collected material was suspended in PBS (0.5 mL) for subsequent analysis.

- Laboratory Procedures

Salivary pH Measurement: pH was quantified immediately post-collection using an ion-selective analyzer (STARTER 3100 BENCH), calibrated with certified buffer solutions. Triplicate measurements ensured reproducibility.

Nickel Quantification: Nickel concentrations were determined via graphite furnace atomic absorption spectrophotometry (Thermo Scientific iCE 3000) at 232 nm (20,21). Sample-specific pretreatments included:

• Saliva: Centrifugation (10,000 ×g, 10 min) and dilution with 0.5% nitric acid.

• Biofilm: Homogenization (30 sec), sonication (15 min), centrifugation (12,000 ×g, 15 min), and acid resuspension.

• Gingival Tissue: Digestion with proteinase K (2 h, 56°C), enzyme inactivation (95°C, 10 min), and centrifugation. The assay’s detection and quantification limits were 0.1 μg/L and 0.3 μg/L, respectively (22,23).

Statistical Analysis

All statistical analyses were performed using SPSS v25 (IBM Corp.), with non-parametric methods selected due to the non-normal distribution of nickel concentration data, as confirmed by Kolmogorov-Smirnov and Shapiro-Wilk tests. Descriptive statistics summarized the data as means ± standard deviations or medians with interquartile ranges, depending on distribution characteristics. For longitudinal comparisons across the three time points (T0, T1, T2), Friedman’s test for repeated measures was applied, with post-hoc pairwise analyses conducted using Wilcoxon signed-rank tests and Bonferroni correction to account for multiple comparisons. Differences in nickel accumulation between biospaces (saliva, biofilm, gingival tissue) were assessed via Kruskal-Wallis tests, supplemented by Mann-Whitney U tests for specific contrasts. The relationship between salivary pH and nickel concentrations was evaluated using Spearman’s rank correlation coefficient (ρ). To identify independent predictors of nickel accumulation, a stepwise multiple linear regression model was constructed, incorporating variables such as treatment duration, baseline nickel levels, and salivary pH. The model’s assumptions were rigorously verified, including linearity (residual plots), normality of residuals (Q-Q plots), homoscedasticity (Breusch-Pagan test), and absence of multicollinearity (variance inflation factors < 5). A two-tailed significance level of α = 0.05 was maintained throughout, with *p-value*s adjusted for false discovery rate using the Benjamini-Hochberg procedure. Sensitivity analyses restricted to participants with complete follow-up data confirmed the robustness of the primary findings.

## Results

- Participant Demographics and Baseline Characteristics

The study retained 120 of 135 initially screened participants (88.9% completion rate), comprising 71 females (59.2%) and 49 males (40.8%), with a mean age of 24.6 ± 7.5 years. Malocclusion distribution followed Angle’s classification: Class I (35.8%), Class II (43.4%), and Class III (20.8%). Baseline oral health metrics included a median plaque index of 26.5% (IQR: 18.2–37.8) and unstimulated salivary flow rate of 0.48 mL/min (IQR: 0.39–0.63), indicating representative physiological variation ( Table 1). Normality testing confirmed non-parametric distributions for nickel concentrations across all biospaces (saliva, biofilm, gingiva; *p* < 0.01), warranting non-parametric statistical methods.

- Longitudinal Patterns of Nickel Accumulation Across Oral Biospaces

Longitudinal analysis revealed distinct temporal patterns of nickel accumulation in each biospace, with statistically significant variations over time (all *p* < 0.001, Friedman’s test): biofilm (χ² = 74.82), gingival tissue (χ² = 42.19), and saliva (χ² = 59.36).

**Figure 1:**
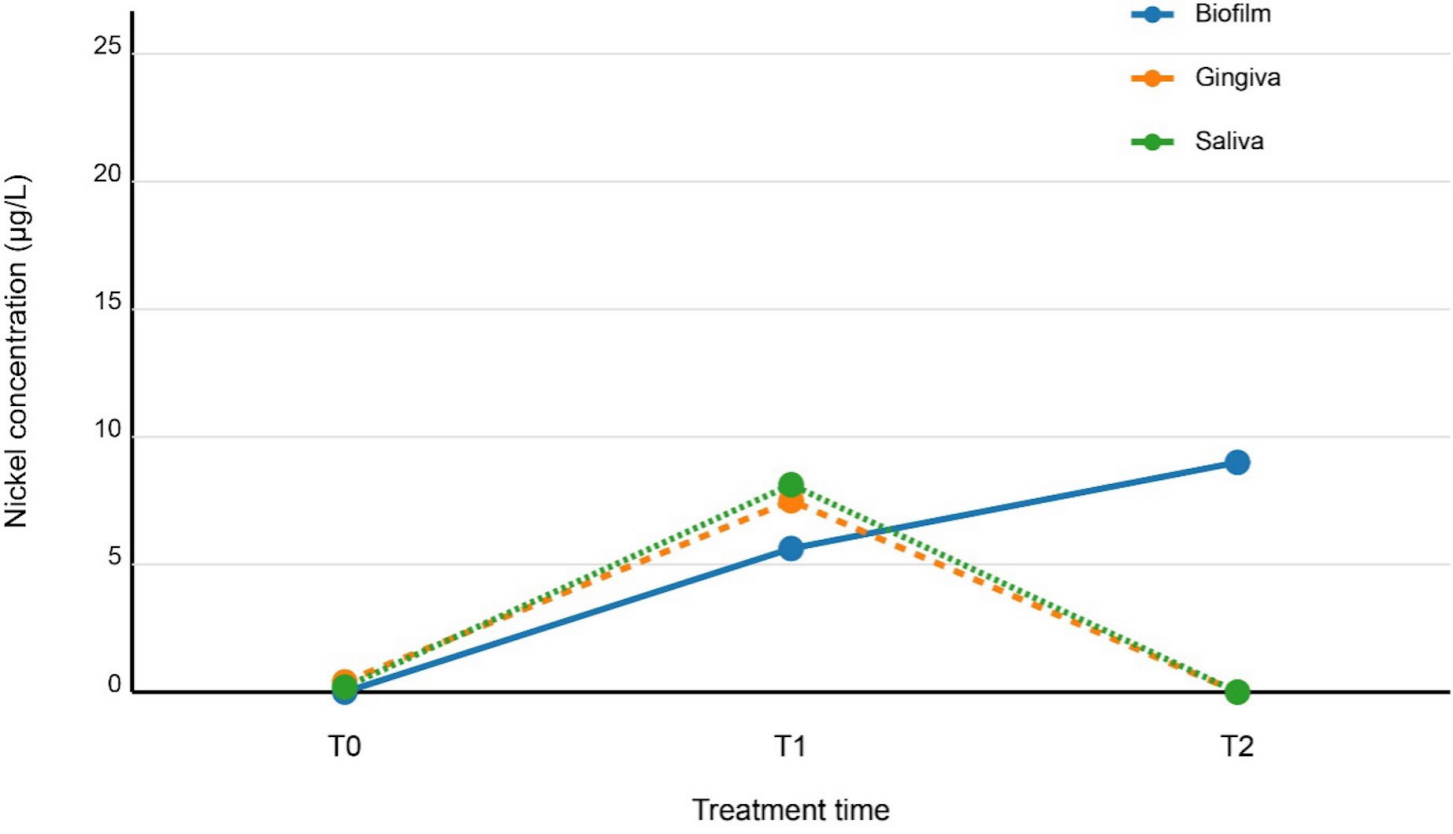
Temporal trends of nickel concentrations in biofilm, gingiva, and saliva over 12 months of orthodontic treatment.

Biofilm exhibited progressive nickel accumulation, with median concentrations increasing from 0.0008 µg/L (IQR: 1.2102) at baseline (T0) to 21.5833 µg/L (IQR: 26.6630) at T2. Pairwise comparisons confirmed significant increases between all intervals (T0–T1: Z = −7.24; T1–T2: Z = −5.18; T0–T2: Z = −8.92; all *p* < 0.001) ( Table 2, Fig. 1).

Gingival tissue displayed a biphasic trend: a sharp rise from T0 (0.9538 µg/L; IQR: 0.9194) to T1 (18.0535 µg/L; IQR: 20.2113; Z = −7.65, *p* < 0.001), followed by a decline to near-baseline levels at T2 (0.0024 µg/L; IQR: 14.5824; T1–T2: Z = −6.17, *p* < 0.001). Net change from T0–T2 showed a slight but significant decrease (Z = −4.78, *p* < 0.001) ( Table 2, Fig. 1).

Saliva mirrored gingival dynamics, peaking at T1 (19.5069 µg/L; IQR: 15.8056) after an initial rise from T0 (0.4777 µg/L; IQR: 0.5703; Z = −8.39, *p* < 0.001), then decreasing by T2 (0.0020 µg/L; IQR: 20.7990; Z = −7.12, *p* < 0.001). The overall T0–T2 reduction was significant (Z = −4.96, *p* < 0.001) ( Table 2, Fig. 1).

Salivary pH remained sTable throughout the study (medians: T0 = 7.21, T1 = 7.36, T2 = 7.48; χ² = 4.06, *p* = 0.131), with no clinically relevant fluctuations ( Table 2).

- Comparative Nickel Distribution Across Oral Biospaces and pH Correlations

Analysis of nickel distribution patterns revealed significant variations among oral biospaces that evolved throughout treatment. At baseline (T0), Kruskal-Wallis testing demonstrated substantial differences in nickel concentrations (H=68.37, *p*<0.001), with a distinct hierarchy: gingival tissue showed the highest levels (median=0.954 µg/L), followed by saliva (0.478 µg/L; U=2476.5, *p*=0.006 vs gingiva), and biofilm containing the lowest concentrations (0.001 µg/L; U=1824.0, *p*<0.001 vs gingiva). Saliva concentrations were also significantly elevated compared to biofilm (U=2083.5, *p*<0.001).

By 6 months (T1), nickel levels increased markedly across all biospaces but without significant inter-biospace differences (H=3.27, *p*=0.195), indicating a period of homogeneous ion distribution. This pattern shifted again by T2, when significant differences reemerged (H=64.92, *p*<0.001). At this final stage, biofilm accumulated substantially higher nickel concentrations (21.583 µg/L) compared to both gingiva (0.002 µg/L; U=1972.5, *p*<0.001) and saliva (0.002 µg/L; U=2137.0, *p*<0.001), while gingival and salivary levels no longer differed (U=5534.0, *p*=0.427).

**Figure 2:**
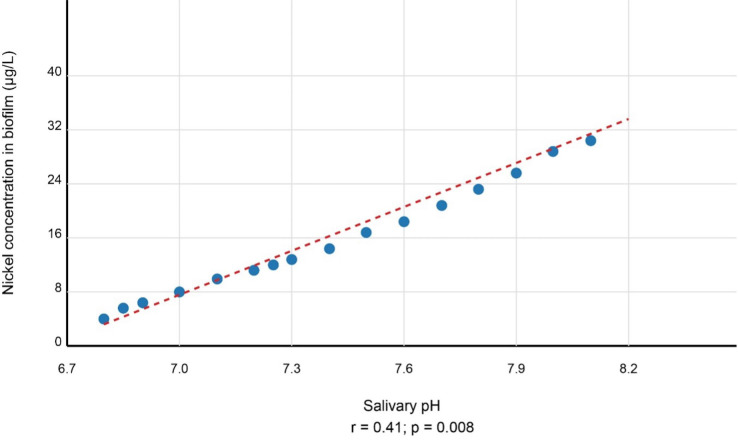
Progressive strengthening of correlation between salivary pH and nickel concentration in oral biofilm over 12 months of orthodontic treatment. The association evolved from non-significant at baseline (T0) to moderate positive correlation at study completion (T2).

A notable temporal evolution emerged in the relationship between salivary pH and nickel accumulation. While no significant correlation existed at baseline (r=0.17, *p*=0.112), a weak but significant positive association developed by T1 (r=0.25, *p*=0.047), strengthening to a moderate correlation by T2 (r=0.41, *p*=0.008) specifically for biofilm nickel concentrations (Fig. 2). In contrast, gingival nickel levels showed no significant pH correlations at any timepoint. Salivary nickel demonstrated weak but consistent pH-dependent associations at T1 (r=0.26, *p*=0.041) and T2 (r=0.28, *p*=0.031).

- Predictors of Nickel Accumulation: Multivariate Regression Analysis

Multiple linear regression models identified distinct predictive factors for nickel accumulation in each oral biosphere at treatment completion (T2), with all models demonstrating significant predictive power (all *p*<0.001). The analysis revealed three key patterns of nickel deposition influenced by different combinations of clinical and biological variables ( Table 3).

For biofilm, the strongest model (adjusted R²=0.62) identified treatment duration as the predominant predictor (β=0.67, 95%CI:0.48-0.86, *p*<0.001), accounting for the majority of explained variance. Two secondary modulators emerged: salivary pH showed a positive association (β=0.22, *p*=0.027), while patient age demonstrated an inverse relationship (β=-0.19, *p*=0.041). Notably, biofilm accumulation was pH-sensitive but independent of oral hygiene status (plaque index β=0.15, *p*=0.126).

The gingival tissue model (adjusted R²=0.48) revealed different dynamics, with treatment duration (β=0.56, *p*<0.001) and plaque index (β=0.31, *p*=0.008) as the only significant predictors. Unlike biofilm, gingival nickel accumulation was unaffected by salivary pH (β=0.14, *p*=0.192) or demographic factors, suggesting a primarily mechanical deposition pathway influenced by local hygiene.

Salivary nickel concentrations (adjusted R²=0.52) showed yet another pattern, with significant contributions from treatment duration (β=0.63, *p*<0.001), salivary pH (β=0.24, *p*=0.019), and salivary flow rate (β=-0.22, *p*=0.031). The inverse relationship with flow rate suggests dilution effects may modulate free ion concentrations in oral fluids.

All models demonstrated appropriate diagnostic metrics (VIF<1.4 for all variables, Durbin-Watson=1.92 for biofilm), with residual analyses confirming normality (*p*=0.318) and homoscedasticity (*p*=0.425). Sensitivity analysis restricted to participants with complete follow-up (*n*=108) yielded virtually identical coefficients, confirming model robustness.

## Discussion

This 12-month prospective cohort study provides novel insights into the spatiotemporal dynamics of nickel bioaccumulation during fixed orthodontic treatment, revealing three key findings: 1) distinct accumulation patterns across oral biospaces, 2) biofilm’s emerging role as the dominant long-term nickel reservoir, and 3) pH-dependent modulation of nickel retention. These results significantly advance our understanding of orthodontic biomaterial-oral environment interactions and have important clinical implications.

The observed temporal patterns (Fig. 1) demonstrate fundamental differences in nickel handling between biospaces. Biofilm showed progressive accumulation (0.0008→21.5833 µg/L), while gingival and salivary concentrations followed biphasic kinetics - peaking at 6 months (18.0535 and 19.5069 µg/L respectively) before returning near baseline. This pattern extends previous reports of initial release phases [9,11] by revealing subsequent redistribution, suggesting active biological regulation. The biofilm trajectory likely reflects microbial metal sequestration through: (i) specific uptake systems in acidogenic species [17], (ii) extracellular polymeric substance (EPS) metal-binding capacity [19], and (iii) pH-microenvironment facilitated precipitation [22]. Conversely, the gingival/salivary decline may indicate activation of host detoxification mechanisms, including metallothionein upregulation [20] and salivary protein complexation [22,23], highlighting the mouth’s remarkable metal homeostasis capacity.

The spatial redistribution is particularly noteworthy. While gingiva initially showed highest concentrations (0.954 µg/L at T0, reflecting environmental exposure), biofilm became the dominant reservoir by T2 (21.583 µg/L, 1000× higher than other biospaces). This shift suggests biofilm actively modulates nickel bioavailability, potentially protecting host tissues through sequestration, a “sacrificial sink” phenomenon. This finding aligns with but substantially extends Urbutytė’s plaque observations [20], demonstrating biofilm’s cumulative rather than transient retention capacity. Clinically, this positions oral biofilm as both an exposure biomarker and potential intervention target through antimicrobial/chelating strategies [24-26].

Our pH correlation findings (Fig. 2) provide mechanistic insights, showing progressively strengthening biofilm-nickel associations (r=0.17→0.41). This temporal amplification suggests even physiologically normal pH variations (7.21-7.48) exert cumulative effects on alloy corrosion - likely through: 1) passivation layer destabilization [2], 2) enhanced EPS metal-binding at higher pH [19], and 3) pH-dependent microbial metabolism shifts [17]. The specificity to biofilm (absent in gingiva) implies microbiome-mediated pH effects, supporting recent *in vitro* corrosion-microbiome studies [27,28].

The multivariate regression analysis revealed distinct predictive patterns for nickel accumulation across oral biospaces, with treatment duration emerging as the dominant predictor in all models. For biofilm accumulation, our strongest model (adjusted R²=0.62) showed treatment time (β=0.67, *p*<0.001) accounting for most variance, supplemented by significant contributions from salivary pH (β=0.22, *p*=0.027) and an inverse association with patient age (β=-0.19, *p*=0.041). Gingival tissue followed a different pattern, where treatment duration (β=0.56, *p*<0.001) and plaque index (β=0.31, *p*=0.008) were the sole significant predictors, suggesting mechanical deposition processes influenced by local hygiene rather than systemic factors. Salivary nickel concentrations presented yet another distinct profile, with treatment time (β=0.63, *p*<0.001), salivary pH (β=0.24, *p*=0.019), and flow rate (β=-0.22, *p*=0.031) all contributing significantly, the latter indicating potential dilution effects. All models demonstrated excellent diagnostic parameters with variance inflation factors <1.4 and appropriate residual distributions, while sensitivity analysis confirmed robust coefficients when restricted to participants with complete follow-up (*n*=108). These findings collectively highlight how nickel distribution mechanisms vary fundamentally between oral compartments, from biofilm’s pH-sensitive accumulation to saliva’s flow-dependent clearance patterns, providing a nuanced understanding of exposure dynamics during orthodontic treatment.

While this study provides comprehensive longitudinal data on nickel bioaccumulation dynamics, several limitations should be considered when interpreting the results. The non-randomized design, while appropriate for observational cohort studies, limits our ability to make causal inferences about specific alloy effects. Our total nickel quantification approach, though sensitive, did not examine chemical speciation which could provide important insights into bioavailability and toxicity profiles. Additionally, while we identified significant pH correlations with biofilm accumulation, we did not characterize the oral microbiome variations that may mediate these relationships. Perhaps most notably, despite nickel’s well-documented allergenic potential, we did not correlate our exposure data with clinical hypersensitivity manifestations, a connection that would significantly strengthen the clinical relevance of our findings. These limitations simultaneously highlight valuable opportunities for future investigation, particularly studies incorporating randomized alloy comparisons, advanced analytical techniques for nickel speciation, and comprehensive microbiome-metallome correlations.

Building on these findings, several critical research directions emerge as particularly promising. Randomized controlled trials comparing different orthodontic alloys would help establish material-specific bioaccumulation profiles. The application of synchrotron-based X-ray absorption spectroscopy could reveal nickel speciation patterns and their relationship to bioavailability. Comprehensive metagenomic characterization of the oral microbiome, coupled with metallomic analysis, would help elucidate the microbial contributions to pH-dependent nickel retention that our study suggests. At the tissue level, evaluating established biomarkers of cellular stress (such as 8-hydroxy-2’-deoxyguanosine for oxidative DNA damage or matrix metalloproteinase-9 for tissue remodeling) could bridge the gap between nickel exposure and biological impact. Most importantly, prospective studies correlating these exposure measures with clinically documented hypersensitivity reactions would substantially advance our ability to predict and prevent adverse outcomes in orthodontic patients.

The current findings suggest several practical applications that could enhance clinical practice. The identification of oral biofilm as the primary long-term nickel reservoir supports its use as a non-invasive exposure biomarker, potentially allowing clinicians to identify high-risk patients through simple plaque sampling. The characteristic peak exposure observed at 6 months post-placement defines a critical prevention window when targeted interventions, such as enhanced plaque control or chelating mouth rinses, may be most effective. Our demonstration of pH-modulated nickel retention suggests that pH-modified oral care products could be developed to reduce metal bioavailability. Most fundamentally, the overwhelming predictive power of treatment duration across all models strongly supports efforts to minimize unnecessary treatment time through efficient biomechanics and case selection. Together, these insights provide an evidence base for developing comprehensive strategies to monitor and mitigate nickel exposure during orthodontic therapy.

## Conclusions

This longitudinal study demonstrates that nickel ions accumulate in distinct temporal patterns across oral biospaces during orthodontic treatment: progressive accumulation in biofilm versus biphasic kinetics (peak-then-decline) in gingival tissue and saliva. Multivariate analysis identified treatment duration as the strongest predictor across all compartments (β=0.56–0.67), with additional modulation by salivary pH in biofilm (β=0.22) and saliva (β=0.24), and an inverse association with age in biofilm (β=−0.19). These findings reveal fundamental differences in nickel handling mechanisms among oral biospaces.

The study establishes oral biofilm as the dominant long-term nickel reservoir, reaching concentrations 1000× higher than other biospaces by 12 months (21.58 vs 0.002 µg/L). This preferential accumulation, coupled with its pH-dependent behavior (r=0.41 at T2), suggests biofilm may serve a protective role by sequestering ions from host tissues—a phenomenon with important clinical implications. First, it positions biofilm as a practical biomarker for exposure monitoring. Second, it highlights the potential for nickel exposure reduction through biofilm-targeted strategies, including optimized oral hygiene protocols and pH-modulating agents.

The progressively strengthening pH-nickel correlations (r=0.17→0.41) underscore how subtle microenvironmental changes influence corrosion dynamics over time. These results provide an evidence base for two key advancements: 1) development of corrosion-resistant orthodontic biomaterials with stable passivation layers, and 2) clinical monitoring systems leveraging biofilm analysis to identify high-risk patients. Future research should explore whether reducing biofilm nickel loads through targeted interventions decreases the incidence of nickel hypersensitivity reactions.

## Figures and Tables

**Table 1:** Baseline Sociodemographic and Clinical Characteristics of Study Participants (n= 120).

Characteristic	n	%	Mean ± SD	Median [IQR]
Gender				
Male	49	40.8		
Female	71	59.2		
Age (years)			24.63 ± 7.5	23.0 [18.0-29.5]
Malocclusion type				
Class I	43	35.8		
Class II	52	43.4		
Class III	25	20.8		
Plaque index (%)			28.4 ± 12.7	26.5 [18.2-37.8]
Salivary flow (ml/min)			0.52 ± 0.18	0.48 [0.39-0.63]

**Table 2:** Nickel Concentrations (µg/L) and Salivary pH Across Study Time Points.

Time	Variable	Median	IQR	p-value
T0	Biofilm	0.000831	1.210199	<0.001*
	Gingiva	0.953828	0.919357	<0.001*
	Saliva	0.477747	0.570258	<0.001*
	Salivary pH	7.210	0.810	0.131
T1	Biofilm	13.499500	19.420568	
	Gingiva	18.053500	20.211301	
	Saliva	19.506945	15.805555	
	Salivary pH	7.355	1.068	
T2	Biofilm	21.583333	26.662975	
	Gingiva	0.002425	14.582352	
	Saliva	0.002000	20.799000	
	Salivary pH	7.480	0.925	

*p-value corresponds to Friedman’s test for global comparison between the three times †Significant differences in post-hoc pairwise comparisons (*p* <0.05 with Bonferroni correction)

**Table 3:** Multivariate predictors of nickel concentrations by oral biosphere at treatment completion (T2).

Biospace / Predictor	Standardized coefficient	95% CI	p-value	VIF	Adjusted R² (model)
Biofilm (T2)					0.62
Treatment time	0.67	0.48 to 0.86	<0.001*	1.09	
Salivary pH	0.22	0.03 to 0.41	0.027*	1.17	
Age	-0.19	-0.37 to -0.01	0.041*	1.12	
Sex (ref: female)	0.09	-0.11 to 0.29	0.382	1.07	
Plaque index	0.15	-0.04 to 0.34	0.126	1.38	
Gingiva (T2)					0.48
Treatment time	0.56	0.38 to 0.74	<0.001*	1.12	
Plaque index	0.31	0.12 to 0.50	0.008*	1.24	
Salivary pH	0.14	-0.07 to 0.35	0.192	1.06	
Age	-0.11	-0.30 to 0.08	0.258	1.18	
Saliva (T2)					0.52
Treatment time	0.63	0.44 to 0.82	<0.001*	1.11	
Salivary pH	0.24	0.05 to 0.43	0.019*	1.08	
Salivary flow	-0.22	-0.41 to -0.03	0.031*	1.14	
Age	-0.14	-0.33 to 0.05	0.146	1.09	

*p-value <0.05. VIF: Variance Inflation Factor. Significant F test (*p* <0.001) for all models. Biofilm model diagnostics: Durbin-Watson=1.92; residual normality test p=0.318; homoscedasticity test p=0.425

## Data Availability

The datasets used and/or analyzed during the current study are available from the corresponding author.
